# Visualization and identification of health space, based on personalized molecular phenotype and treatment response to relevant underlying biological processes

**DOI:** 10.1186/1755-8794-5-1

**Published:** 2012-01-06

**Authors:** Jildau Bouwman, Jack TWE Vogels, Suzan Wopereis, Carina M Rubingh, Sabina Bijlsma, Ben van Ommen

**Affiliations:** 1 Microbiology and System Biology, TNO, Zeist, The Netherlands; 2 Quality and Safety, TNO, Zeist, The Netherlands

## Abstract

**Background:**

Being able to visualize multivariate biological treatment effects can be insightful. However the axes in visualizations are often solely defined by variation and thus have no biological meaning. This makes the effects of treatment difficult to interpret.

**Methods:**

A statistical visualization method is presented, which analyses and visualizes the effects of treatment in individual subjects. The visualization is based on predefined biological processes as determined by systems-biological datasets (metabolomics proteomics and transcriptomics). This allows one to evaluate biological effects depending on shifts of either groups or subjects in the space predefined by the axes, which illustrate specific biological processes. We built validated multivariate models for each axis to represent several biological processes. In this space each subject has his or her own score on each axis/process, indicating to which extent the treatment affects the related process.

**Results:**

The health space model was applied to visualize the effects of a nutritional intervention, with the goal of applying diet to improve health. The model was therefore named the 'health space' model. The 36 study subjects received a 5-week dietary intervention containing several anti-inflammatory ingredients. Plasma concentrations of 79 proteins and 145 metabolites were quantified prior to and after treatment. The principal processes modulated by the intervention were oxidative stress, inflammation, and metabolism. These processes formed the axes of the 'health space'. The approach distinguished the treated and untreated groups, as well as two different response subgroups. One subgroup reacted mainly by modulating its metabolic stress profile, while a second subgroup showed a specific inflammatory and oxidative response to treatment.

**Conclusions:**

The 'health space' model allows visualization of multiple results and to interpret them. The model presents treatment group effects, subgroups and individual responses.

## Background

Emerging technologies in biological research can provide an enormous wealth of data. At the same time, researchers are challenged to interpret this data in an integrated and meaningful manner. Multiple parameters describe processes and interactions between processes. Multivariate statistical approaches like pattern recognition are applied in the data analysis. Many multivariate visualization and classification tools have been developed [1,2], both 'unsupervised' and 'supervised' (discriminating on treatment group). For example, principle component analysis [3,4] (an unsupervised method) uses the largest variation in the data to define a first axis (principle component). This method has been shown to help structure and interpret experiments [5]. From a biological interpretation viewpoint, the drawback of this method is that the axes are defined only by variation and therefore have no biological meaning. Another statistical method that takes the information on groups into account is the Partial Least Squares Discrimination Analysis [6] (a supervised method). In this method, the first axis is defined based on the line that best separates the defined groups. However, this method also does not provide useful information about the underlying biological processes. Additionally, most of these statistical methods only separate subjects into groups and do not provide information on the status of individual subjects within the group. Separating, classifying and visualizing data based on *a priori *defined biological processes would greatly facilitate the biological interpretation of the data. Examples of methods that do so are the Metabolite Set Enrichment Analysis (MSEA), Gene Set Enrichment Analysis (GSEA) and GSEA based cluster analysis [7,8]. These methods, however, only focus on group averages.

Since extensive phenotyping enables researchers to accurately describe multiple aspects of a biological response to a treatment, it is possible to apply these results towards a personalized health strategy instead of (or on top of) strategies based on group averages [9].

We have developed an analysis and visualization method, named the 'health space', which projects subjects' health status in a multidimensional space, based on predefined multivariate parameterization of the axes. This allows researchers to analyze responses according to the underlying biological processes, defining the parameterization in a biologically meaningful manner. As demonstrator case, we applied this method to a recently published study [10] in which relatively healthy overweight subjects were treated in a cross-over design with a diet containing n-3 fatty acids, Epigallocatechin gallate (EGCG), Vitamin E, Vitamin C, resveratrol, and tomato extract. The diet was designed to improve the subjects' health status on the parameters inflammation, oxidation and metabolism. In this case, the biological processes oxidative stress, metabolic stress and inflammation were used to define a 'health space'. The plasma concentrations of 47 clinical chemistry variables, 79 proteins and 274 metabolites before and after a five week treatment period were analyzed, separately and together with the transcriptome of Peripheral Blood Mononuclear Cells (PBMC) (10812 transcripts). Only the significantly changed parameters were used (including 7 parameters for oxidation, 18 for inflammation and 115 for metabolism) to define the 'health space'.

## Methods

### Study design, execution and analytical methods

The study has been described in detail [10]. In short, 33 males (BMI 25.5 - 35 kg/m2 and CRP 1-10 mg/L) were treated for 5 weeks with a dietary mix (resveratrol, green tea extract, alpha-tocopherol, vitamin C, omega-3 polyunsaturated fatty acids, and tomato extract) in a double blind placebo controlled cross-over design. Measurements performed on plasma were established biomarkers (n = 47), lipidome by LC-MS (n = 108), metabolome by GCMS (145) and multiplex quantification of 79 plasma proteins. Transcriptomics was performed on PBMC using Affymetrix arrays (10812 transcripts).

### Axis definition

The selection of the 'overarching processes' oxidation, metabolism and inflammation was adopted from the human study [10]. The selection of significant affected parameters and the grouping of these molecules in the three processes are described in this reference (see [10], Additional file 1). The mean value over time was calculated for parameters that showed significance after multiple testing. The axes contained both the t0 value and the mean value over time for each parameter when both were found to differ significantly between the dietary mix group and the control group.

### Statistics

'Health' or 'healthy state' was defined as the average of the dietary mix group. This point was defined as the origin of the health space. A separate autoscaled double cross validated PLS-DA model [6] was built for each of the three processes, based on significantly changed clinical chemistry, metabolite and protein parameters per process (Additional file 1). To allow comparison of the scores within a process and prevent quantitative comparisons between processes, the samples were scaled such that the PLS-DA scores from the subjects treated with the dietary mix group have mean 0 (zero) and the scores from the control group have mean 1 (one) for all three axes. This was performed for each individual score for each process by subtracting the mean score of the anti-inflammatory-dietary mix group and dividing by the distance between the mean score of the dietary mix group and the mean score of the placebo group. Scaling was performed simultaneously on all scores (including control and treated data points) of each PLS-DA model. The overall result of this treatment was visualized by plotting the scaled PLS-DA prediction scores in a 3D-plot where the three processes span the axes. The axes are statistically and biologically interdependent and are therefore not orthogonal, but are visualized in a 3D plot. The formation of clusters of samples inside this 3D space was analyzed by hierarchical clustering (based on Euclidean distance, average linkage and no additional scaling using the Matlab Bioinformatics Toolbox V3.2 Matlab™ [11]).

A two-way ANOVA was applied to investigate the interaction between cluster groups obtained from hierarchical clustering and treatment. In the case of no significant interaction, the main effects were studied. A false discovery rate correction was applied on significant p-values using the Benjamini and Hochberg method [12]. The null hypothesis (no effect) was rejected at the alpha = 0.05 significance level. The SAS statistical software package (versions 8.2 and 9.1; SAS Institute Inc, Cary, NC) was used for univariate statistical analysis.

## Results

### Constructing the model

**Figure 1:**
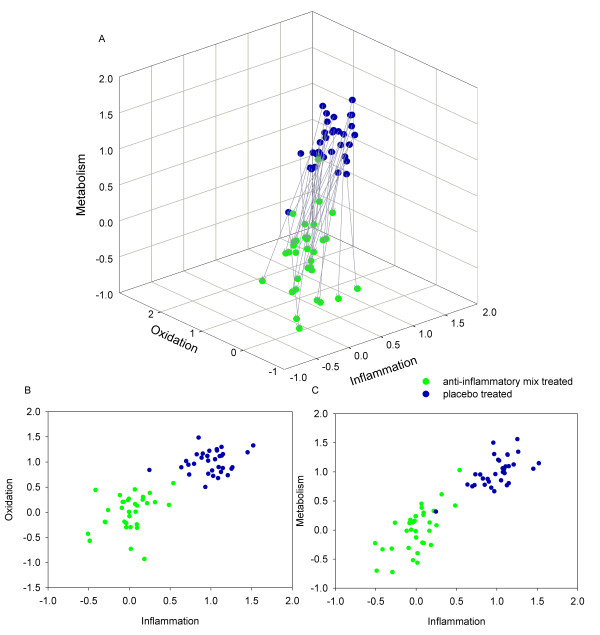
**Health space model with dietary compounds**. Double-cross validated PLS-DA model for each process on an axis and based on significantly changed parameters in the process (metabolism; n = 115, inflammation; n = 18; oxidation; n = 7). Note that each process contained a different number of parameters. The model is scaled around 0 for the treated group and 1 for the untreated group. A: 3-D representation B&C: 2-D representation.

The plasma clinical chemistry, metabolomics and proteomics results of a human nutritional intervention study were visualized in a 3-dimensional space (Figure 1), where the three axes represented the 'overarching processes' oxidation, metabolism and inflammation. These processes were shown to be affected by the treatment [10]. For these processes p = 7, 115 and 18 parameters respectively, changed significantly (p < 0.05) due to the intervention (Additional file 1). Each subject is represented in this 3-D space (both prior to and after treatment), based on the unique values of these 140 metabolites and proteins. The method is based on the construction of three PLS-DA models, one for each of the above processes. A comparison between scores within a PLS-DA model is meaningful, but scores can not directly be compared between PLS-DA models. In order to compare the scores per process, the scores of the three models were subsequently scaled around zero (the average of the dietary-mix group) and one (average of the placebo group). The difference between 0 and 1 was used to separate out relatively healthy subjects from less healthy subjects, assuming that people become healthier using the anti-inflammatory mix. This is further described in the methods section.

Analysis and visualization of the effects of the anti-inflammatory mix showed a separation between the treated and control groups, suggesting an average treatment effect. This is logical, since the axes were constructed using all parameters that changed significantly for the average study group. The unique feature of this visualization model, however, is that the changes were visualized and directly correlated to the relevant biological processes (metabolism, oxidation and inflammation). Overall, a treatment related shift was observed along the predefined inflammation and metabolism axes, and to a lesser extent along the oxidation axis. This is emphasized by the 2-D projections in Figure 1. Note that the process oxidation contained fewer parameters (Additional file 1).

In addition to the group treatment effect, strong differences in inter-individual responses were visualized (i.e., the length of the vector is different, but the direction is similar).

### Dietary compounds have a marked effect on the response

As the health space model is partly based on metabolic profiles, plasma metabolites derived from the dietary intervention itself might interfere with the division into groups. These metabolites would thus report on the diet effect in plasma rather than on the resulting change in metabolism or related health status. Therefore, we rebuilt the health space without the dietary metabolites (omega-3 PUFAs, 2,3,4,-trihydroxybutanoic acid and vitamin E).

**Figure 2:**
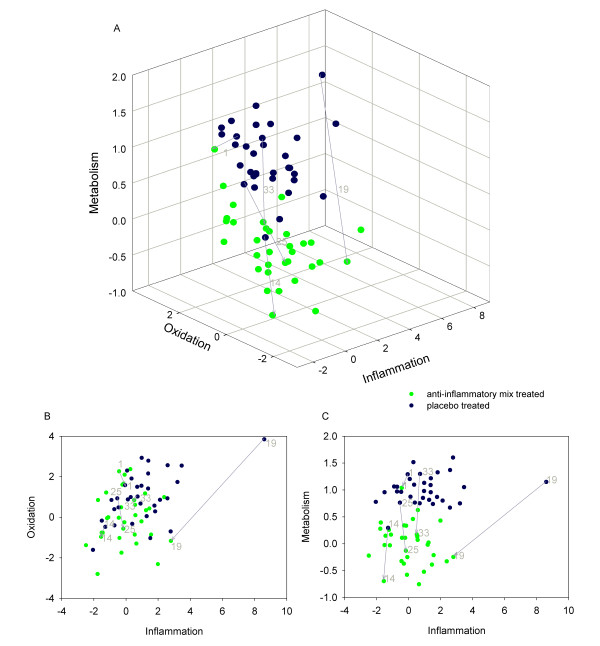
**Health space model without dietary compounds**. Double-cross validated PLS-DA model for each process on an axis and based on significantly changed parameters in the process without the dietary compounds (metabolism; n = 114, inflammation; n = 15; oxidation; n = 5). Note that each process contained a different number of parameters. The model is scaled around 0 for the treated group and 1 for the untreated group. A: 3-D representation B&C: 2-D representation.

**Figure 3:**
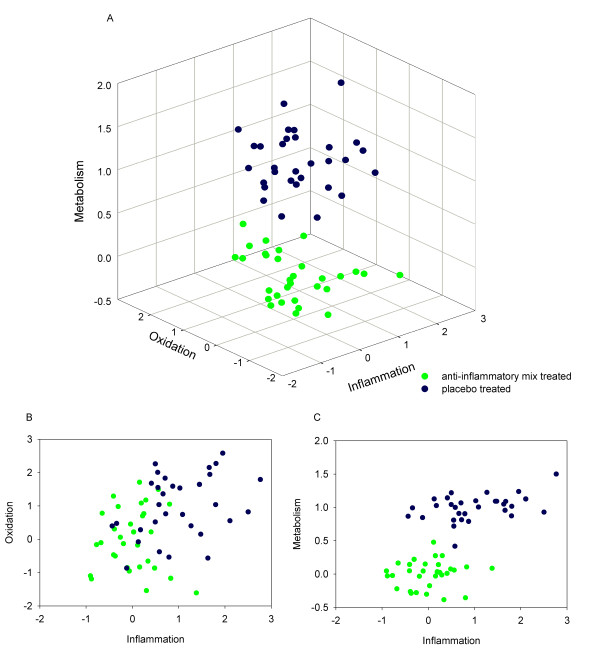
**Health space model including transcriptomics data**. Health space including transcriptomics data. Double-cross validated PLS-DA model was built for each process on an axis based on significantly changed parameters in the process without the dietary compounds (metabolism; n = 154, inflammation; n = 37; oxidation; n = 10). A: 3-D representation B&C: 2-D representation.

After excluding the dietary metabolites, we built two models, one using metabolomics and proteomics data (Figure 2) and a second additionally including PBMC transcriptomics data (Figure 3). Both models made it possible to distinguish the placebo- and dietary intervention groups, showing that the health space could be used for different data types. However, since the classification of transcripts in the selected functional groups (metabolism, oxidation and inflammation) requires improvement and the selection of the processes oxidation, inflammation and metabolism as axes is based on proteomics and metabolomics data [10], we further focus on the health space based only on significantly changed proteins and metabolites.

**Figure 4:**
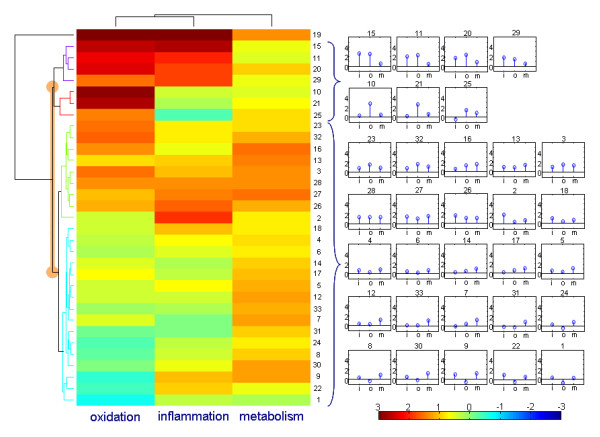
**Hierarchical clustering of individual response**. Hierarchical clustering of individual response of the subjects on the three processes. Every person has his individual change in all three processes within the 'health space'. The individual change for every subject is indicated per process (fig 3B) and clustered according to the resulting score pattern (fig 3A). Person 19 is an outlier and not used for further analysis. Two main groups can be distinguished.

In the model, based on metabolomics and proteomics data and excluding the dietary metabolites, it was possible to distinguish the placebo group from the dietary-mix treated group (Figure 2). Oxidative stress did not significantly contribute to separating the two groups in the new health space. This indicated that the dietary compounds vitamin E and 2,3,4-trihydroxybutanoic acid were the only oxidative parameters that distinguished the placebo from the dietary-mix group in the initial model including dietary compounds. As in the initial health space model, the intensity of the response (length of the vector) differed between subjects. In contrast to the initial model, the contribution of the different processes (direction of the vector) varied between subjects; some people only responded on the inflammation axis, whereas others only responded on the metabolic stress axis. Subject 33 for instance reacted primarily on the metabolism axis. Subject 1 responded on the oxidative axis. Subject 25 showed a combined response on the oxidation and metabolism axes (see also Figure 4). Subject 14 was already within the "improved health area" before the dietary-mix treatment. He had very high C20:5-ChE, C22:6-ChE and poly-unsaturated TG's, which have large loadings in the metabolic PLS-DA model. This is why the fish oil in the dietary-mix scarcely improved Subject 14's metabolic status. Subject 1 responded differently on dietary-mix as compared to the other subjects, as shown by the vector facing in a different direction (Figure 2). This subject had a much higher concentration of plasma VCAM-1 and a lower concentration of M-CSF than average, both after taking the placebo and the dietary mix. He also had relatively high alanine transaminase (ALT) and albumin levels. After taking the placebo, subject 19 showed very different levels of inflammatory parameters compared to the other subjects. The levels of Beta2-microglobulin and uric acid were much higher than average and were even higher after the placebo period than after the dietary-mix period.

### The intervention response of the subjects can be grouped

The individual response was defined as the individual difference between the placebo and the anti-inflammatory mix on the three axes. The individual response to the dietary treatment on the three different processes was clustered in two main groups (Figure 4). Subjects in group 1 mainly showed changes in the inflammation and oxidation parameters. Subjects in group 2 had primarily a metabolic response. Note that oxidation did not separate between the dietary-mix group and the placebo groups, but is important in defining the clusters. The two responder groups we identified did not significantly differ in age, BMI, order of treatment, waist-hip ratio, pre-weight, post-weight or in bodyweight change. The molecules primarily responsible for the separation were C20:5-cholesterol ester, C22:6-cholesterol ester, indole-3-propionic acid, uric acid and arachidonic acid. Indole-3-propionic acid (IPA) had a significant interaction effect. This means that the levels of IPA were affected by the anti-inflammatory treatment in group 1 but not in group 2.

## Discussion

We present a method that analyses and visualizes individuals' health status and their treatment response as derived from multiple, multi-omics parameters of predefined biological processes. Using this method, we showed that an anti-inflammatory dietary intervention lead to significant *average *changes in plasma parameters and that unique individual responses could also be identified. These inter-individual responses were clustered into two distinct subgroups.

The treatment response (i.e., the movement in the health space) did not depend on age, BMI, order of treatment, waist-hip ratio, pre-weight, post-weight or on bodyweight change. The sub-group analysis revealed that individual treatment responses differed from the average group response. For instance, the treatment effect on plasma indole-3-propionic acid concentration was different between the two subgroups. Indole-3-propionic acid is a metabolite that originates from intestinal microbiota [13]. Changes in this metabolite suggest adjustments in size or composition of the intestinal flora after dietary-mix intervention in subjects from responder group 1. The data suggest that the changes in intestinal flora occurred in subjects in group 1, but not in group 2.

The health space also reveals some personalized responses to the dietary intervention. Subject 14, for instance, was already in the improved health group when taking placebo, as a result of relatively high concentrations of fish-oil related parameters. This suggests that this person consumed more fish than average. Additionally, subject 1 responded differently than the other subjects. High plasma ALT and albumin concentrations indicated a biliary obstruction in this subject. A reduction in bile acid release may also reduce the absorption of fatty acids (and lipids and fat soluble vitamins) from the intestine and cause the observed lack in metabolic response. Moreover, this person had a high pro-oxidative-stress response which is known to be related to biliary obstruction [14,15]. Also, the position of subject 19 in the health space was distinctly different than the others. Subject 19 was diagnosed with a malignant tumor (Kahler's disease) two months following the study. This disease may have caused the high levels of VCAM-1 in this subject. The increase of uric acid [16] and beta2-microglobulin [17] after the second period (for this subject the placebo period) indicate the onset of renal dysfunction during the study, which is a known complication of this disease.

The current version of the method builds PLS-DA models for the fold changes of the different parameters involved in the specific processes (scaled to 1). This method presumes that the importance of changes in plasma concentration is relative to the size of the change. This may not necessarily be correct. Certain minor plasma metabolite or protein changes may have large biological consequences, while other large changes may be of little biological relevance. This effect can be taken into account by including additional biological information, e.g. by weighting the components in the different axes. The current model includes only those parameters that significantly changed in the group average analysis. This may underestimate parameters that were affected on an individual basis, i.e. the metabolites and proteins that may be important for personalized health. A model variant may thus include all measured parameters relevant to the selected biological processes, instead of only the significantly changed processes. Another variant being developed includes multivariate feature selection by bootstrapping to select the most significant factors.

In this example of the 'health space' model, we used the processes metabolism, oxidation and inflammation. Some molecules are represented in more than one process (Additional file 1). This way we could take into account that the processes are biologically interdependent.

In the current example, 'health' was simply defined as the average state of metabolism, oxidation and inflammation after the anti- inflammatory intervention (the origin of the model). This demonstrates the applicability of the model. Of course, 'health' should be more accurately defined as a more absolute center (the origin) of the 'health space' model. Furthermore, genetic, life stage and environmental factors influence the chosen biological processes and have an impact on (optimal) health. Other biological processes may be taken into consideration. Multiple studies could be analyzed to more precisely define and refine the health space.

## Conclusions

'Health space' allows scientists to visualize multiple results in a biologically relevant way. This visualization then aids the interpretation of these results. This model presents the effects of treatment, as well as subgroup and individual responses. The practical advantages of this application within a single study are obvious, as this is to our knowledge the first multivariate statistical approach that quantifies and visualizes multiple molecular changes in a biological context and at an individual subject level. Since the model is applicable for any predefined set of biological axes, it can be applied in a wide range of (systems) biology studies.

## Competing interests

All authors are employed by TNO, and none of them reported a conflict of interest related to this article.

## Authors' contributions

JB wrote the paper and gave input regarding the biological aspects of the health space. JTWEV, CMR and SB developed the statistical aspects of the health space and revised the manuscript. SW provided input for the biological aspects of the health space and revised the manuscript. BvO developed the concept and revised the manuscript. All authors read and approved the final manuscript.

## Pre-publication history

The pre-publication history for this paper can be accessed here:

http://www.biomedcentral.com/1755-8794/5/1/prepub

## Supplementary Material

Additional file 1 **Parameters used to define the axes for inflammation, metabolism and oxidation (d = from diet)**. Per parameter p-values and platform type are indicated (derived from Bakker et al.). An empty cell means no data available, ns = not significant, M = metabolomics, P = proteomics, C = clinical data. Click here for file
